# Outcome of urinary bladder cancer after combined therapies

**Published:** 2016

**Authors:** RM Anghel, LN Gales, OG Trifanescu

**Affiliations:** * ”Al Trestioreanu” Institute of Oncology, Bucharest, Romania; ** ”Carol Davila” University of Medicine and Pharmacy, Bucharest, Romania

**Keywords:** bladder cancer, chemotherapy, incidence, radiotherapy, surgery

## Abstract

Abstract

**Rationale:**Urinary bladder cancer is the fourth most common cancer in men and the eighth in women, being an important public health issue.

**Methods:**: Medical files of 155 patients (132M/ 23F) with urinary bladder cancer treated between 2006 and 2012 were retrospectively analyzed. The median age at diagnosis was 65 years (range: 19-85 years). Disease free survival (DFS) for patients with complete tumor resection receiving adjuvant treatment and progression free survival (PFS) for patients with post-operative residual disease was estimated.

**Results:**The distribution of the stage disease was: 50 patients (32.2%) stage II, 47 (30.3%) stage III, 58 (37.4%) stage IV. Radical cystectomy was performed in 56 patients (36.1%), while 99 patients (63.9%) underwent repeated transurethral resection of the urinary bladder tumor (TURBT). Postoperative treatment included multimodal therapy in 47 patients (30.3%) (chemotherapy and external beam radiation), external beam radiation alone in 57 patients (36.8%) and chemotherapy alone (methotrexate, vinblastine, doxorubicin, and cisplatin-MVAC or gemcitabine+platinum) in 51 patients (32.9%). After a median follow-up of 31 months (range: 3-79 months), 51 patients (32.9%) presented local recurrence, 32 patients (21%) distant recurrence (metastases), 10 patients (6.4%) both local and distant recurrence, and 62 patients (40%) were free of disease. The median duration until progression was 27 months.

**Discussion:**Despite the combined therapy approaches, urinary bladder carcinoma remains an aggressive disease, with a high relapse rate. Earlier diagnosis, aggressive radical surgery in intention to cure (cystectomy), and adjuvant multimodal treatment (radiotherapy and chemotherapy) are needed for survival improvement.

## Introduction

Urinary bladder carcinoma is the second most common urologic malignancy [**1**]. It is the fourth most commonly diagnosed malignancy in men and the eighth most common cancer in women [**2**]. Bladder carcinoma represents a spectrum of disease ranging from superficial, well-differentiated disease, which does not significantly affect survival, to highly malignant tumors for which long-term survival may be dismal. Transitional-cell carcinoma, which constitutes the vast majority of bladder cancers (more than 95%), may develop as carcinoma in situ or as invasive carcinoma [**3**]. Presently, radical cystectomy and bilateral lymph node dissection is considered the standard therapeutic option for muscle invasive cancer of the urinary bladder and achieves good local control with minimal perioperative mortality (<1%) [**4**,**5**,**6**]. 

Surgery alone fails to control the cancer of patients who harbor occult systemic disease at time of diagnosis. A multimodality bladder preserving an approach combining transurethral resection (TURBT) with chemoradiation, followed by prompt cystectomy in patients with poorly responding tumors, offers a valid alternative option with long-term survival rates approaching those attained with radical cystectomy [**7**,**8**,**9**].

Metastatic bladder cancer is a chemo-sensitive disease and cisplatin-based therapy is generally considered the standard of care. The combination of methotrexate, vinblastine, doxorubicin, and cisplatin (MVAC) was the first chemotherapy regimen used consistently for bladder carcinoma [**10**,**11**,**12**]. This regimen was associated with significant severe toxicities (grade ≥ 3): nausea and vomiting, neutropenia and febrile neutropenia, mucositis, renal insufficiency, cardiac failure, and death. Thus, a number of new agents such as gemcitabine and taxanes, as well as several platinum-based combinations have been explored. The increasing acceptance and use of gemcitabine + cisplatin (GC) regimen in patients with locally advanced and metastatic bladder cancer is supported by its proven equivalent clinical efficacy compared with MVAC. The 5-year survival data, better tolerability and safety profile of the GC combination, continues to support and strengthen the conclusion that the GC regimen is a safe and effective alternative to MVAC therapy in this patient population [**13**].

The aim of this study was to evaluate the evolution of bladder cancer patients treated in an oncologic center. 

## Methods

Data from medical files of patients with urinary bladder cancer treated in our institute between 2006 and 2012 were retrospectively reviewed. 155 patients were identified with transitional cell carcinoma of the bladder, admitted for oncological treatment. A written informed consent for chemotherapy or radiotherapy treatment was obtained from all patients according to the local policy.

### Patient selection criteria

The eligibility criteria for patients included in the analysis were the following: consecutive patients admitted in our institute for non-surgical treatment (radiotherapy or chemotherapy), having pathology confirmation of the malignancy. 

 At treatment initiation, patients had to be fully recovered after surgery, with an ECOG performance status 0-2, no relevant comorbidities, adequate bone marrow reserve (absolute neutrophils count ≥ 1.5 × 109/ L, platelet count ≥ 100 × 109/ L), normal hepatic (bilirubin level ≤ 1.5 mg/ dL), and renal function (creatinine level ≤ 1.5 mg/ dL), and normal chest X-ray. Cardiac status should have been documented as normal or stable under appropriate treatment, in case of preexisting abnormalities, as defined by the clinical evaluation by a cardiologist and by electrocardiography. 

### Study objective

The main objectives of the analysis were to calculate the disease free survival (DFS) for patients with completed resected tumors who received adjuvant treatment and to determine the progression free survival (PFS) for patients with post-operative residual disease and patients who did not receive a surgical treatment. The acute and late toxicity and tolerability for each treatment modality were ascertained and the intensity of the treatment (both chemotherapy and radiotherapy) was determined in relation with the patients’ compliance. Finally, our results were compared with the available evidences regarding the outcome of bladder cancer patients. 

### Treatment

All the patients initially received a urological evaluation in order to determine if they were able to undergo surgery and if the tumor was resectable, after which they were deemed operable or inoperable. For all inoperable patients, tumor tissue was obtained by transurethral resection of the bladder, in order to have the confirmation of the malignancy. Operable patients were surgically treated by radical cystectomy, partial cystectomy, or transurethral resection of the bladder (TURBT).

 Radiotherapy was administered on the tumor bed or on the tumor volume (for surgically and non-surgically treated patients, respectively), 5 days a week. Four-field external beam radiotherapy was employed by using 10 MEV photons, to a target dose of minimum 4500 cGy. 

All chemotherapy regimens administered were platinum based, using the following combinations: **MVAC** every 28 days [methotrexate (30mg/m2/d on days 1, 14, and 21), vinblastine (3 mg/m2/d on days 1, 14, and 21), adriamycin (30 mg/m2/d on day 2), and cisplatin (70 mg/m2/d on day 2]; **GC** at every 21 days [gemcitabine (1,000-1250 mg/m2/d over 30-60 minutes on days 1 and 8) plus cisplatin (70 mg/m2/d on day 2)].

### Statistical analysis

The statistical analysis was performed by using the SPSS 13.0 for Windows. The following endpoints were analyzed: (i) DFS, defined as the interval between the date of diagnosis and the first recurrence or death by any cause; (ii) PFS, defined as the interval from the date of diagnosis until the date of disease progression or death by any cause. Disease-free and progression-free data were calculated by using the Kaplan-Meyer method. Relevant parameters were studied for influence on survival by univariate analysis while using the log-rank test. A multivariate analysis was performed by using the stepwise cox proportional hazards model to identify independent prognostic factors. Results were considered significant at the 0.05 level.

## Results

Between 2006 and 2012, 155 patients (132M/ 23F) suffering from muscle-invasive (T2 to T4) transitional-cell carcinoma (TCC) of the urinary bladder were admitted for treatment. The patients’ characteristics are listed in **Table 1**. 

**Table 1:**
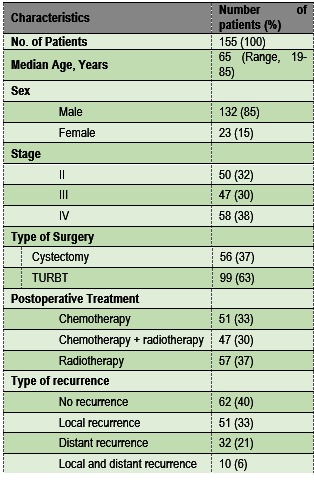
**Table 1**Patient characteristics

The vast majority of patients (85%) were male. The median age at diagnosis was 65 years (range: 19-85). Approximately one quarter of the patients came from rural area, and three quarters from urban area. Most of them (91.6%) were smokers. Hematuria was the presenting symptom in 90% of the cases. Other symptoms at presentation included urinary frequency, urgency dysuria, and ureteral obstruction. Systemic manifestations indicated a metastatic disease and portended a poor prognosis.

The distribution of the stage disease was the following: n=50 patients (32.2%) stage II, n=47 patients (30.3%) stage III, and n= 58 patients (37.4%) stage IV. 

Radical cystectomy was performed in 56 patients (36.1%), while 99 patients (63.9%) underwent repeated transurethral resection of the urinary bladder tumor (TURBT). Postoperative treatment included multimodal therapy in 47 patients (30.3%) (chemotherapy and external beam radiation), external beam radiation alone in 57 patients (36.8%) and chemotherapy alone (MVAC or gemcitabine + platinum) in 51 patients (32.9%). 

Radiotherapy was administered to 104 (67.1%) out of 155 patients, using conformational radiotherapy with a 4-field box technique and daily fractions of 1.8 to 2.0 Gy on 5 consecutive days per week. A median total dose of 55 Gy (range, 2000-6600 cGy) was applied to the bladder and pelvis. The median duration of radiotherapy was 43 days. Radiotherapy was also used as palliative treatment for patients with symptomatic bone metastases. Typical acute radiation-induced side effects, such as transient cystitis and enteritis, were easily managed by symptomatic treatment. Grade 3 or greater hematologic toxicity consisted of leucopenia and anemia and was encountered in 8% of the patients. Other non-hematologic acute adverse reactions were diarrhea in 14% and cystitis in 7% of the patients. 

Regarding chemotherapy, 32% of the patients received gemcitabine plus cisplatin, 11% gemcitabine plus carboplatin, 24% the MVAC regimen, 16% paclitaxel plus carboplatin, 8% cisplatin mono-chemotherapy, and 4% cisplatin concurrently with radiotherapy. Neoadjuvant chemotherapy was administered in 9% of the patients. Afterwards, 7% underwent cystectomy. The median number of chemotherapy cycles administered was 4. Chemotherapy was generally well tolerated, the major toxicity being neutropenia grade 3 and 4 in 11% of the patients and emesis in 32% of the patients. 

**Fig. 1:**
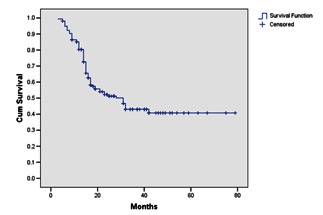
**Fig. 1**Duration until progression in patients with muscular invasive bladder carcinoma

After a median follow-up of 31 months (range: 3-79 months), 51 patients (32.9%) presented local recurrence, 32 patients (21%) distant recurrence (metastases, mainly located at bones and lungs), 10 patients (6.4%) with both local and distant recurrence, and 62 patients (40%) were free of disease. The median duration until progression was 27 months for all patients (**Fig. 1**). The median DFS for patients with stage 3 was only 18 months. For stage 2 patients, the median DFS was not reached at the end of the follow-up, and at 48 months, 58% of the patients were disease free. The PFS for stage 4 was 11 months. 

**Fig. 2 A:**
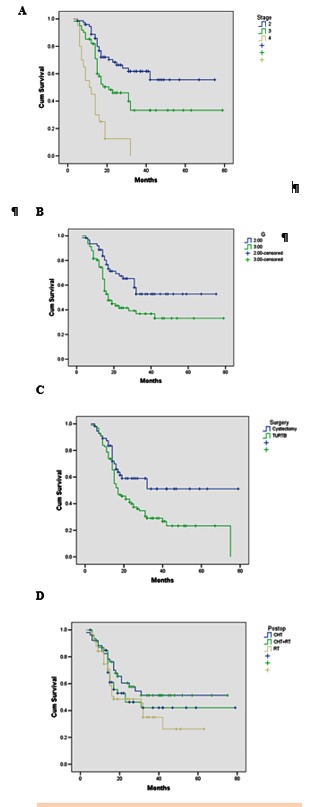
**Fig. 2 A.**Analysis of the outcome of patients with stage 2 vs. stages 3 and 4 (HR=0.34; p<0,001; CI 0.086-0.333 and HR=0.29; p<0.001; CI 0.182-0590, respectively). B. Analysis of the outcome of patients with G2 vs. G3 (HR=0.32; p=0.023; CI 0.182-0.959). C. Analysis of the outcome of the patients with cystectomy vs. TURBT (HR=0.61; p=0.019; CI 0.297-0.905). D. Analysis of the outcome of postoperative treatment of patients (radiotherapy vs. chemotherapy vs. chemotherapy + radiotherapy) NS

The multivariate analysis regarding the outcome of the patients revealed a clear advantage for cystectomy vs. TURBT (HR=0.61; p=0.019; CI 0.397-0.905), for stage 2 vs. stages 3 and 4 (HR=0.34; p<0.001; CI 0.086-0.333 and HR=0.29; p<0.001; CI 0.182-0.590 respectively) and for G2 vs. G3 (HR=0.32; p=0.023; CI 0.182-0.959). There was no statistical difference between the outcomes of different postoperative treatments (**Fig. 2**).

## Discussion

This retrospective analysis showed that the population of bladder cancer patients treated in our institute was highly heterogeneous. 

The characteristics of bladder cancer patients included in our analysis were similar to those of the patients enrolled in many clinical trials [**3**]. 

One of the main aspects that affect the therapeutic strategy is the moment of surgical treatment in relation to the other therapeutic modalities (chemotherapy and radiotherapy). The current recommendation for patients with muscle invasive bladder carcinoma who are able to tolerate extensive surgery is radical cystectomy. If the patient is unwilling to undergo cystectomy (wishing for the preservation of the bladder), a combined modality treatment is advisable; in these situations, one can start with chemotherapy or radiotherapy, followed by a cystoscopy evaluation of the response. 

As the mainstay of treatment for muscle-invasive bladder cancer is surgery, all patients received some form of surgical treatment. What should be taken into account is that only 59% of the patients with stage II and stage III underwent radical cystectomy, while in the rest of cases (including metastatic disease) only transurethral resection of the bladder was performed. The choice of surgery was mainly dictated by the patient’s operability (contingent upon disease stage, age and comorbidities) [**14**]. Thus, when the patients were able to tolerate extended surgery, cystectomy was in general the treatment of choice. Other factors that were taken into consideration were patient preferences and the effectiveness of preoperative treatments (when it was the case). If a cystectomy was not feasible, the patients received transurethral resection of the bladder. 

The disease-free survival of stage II and stage III patients was significantly higher for those treated with cystectomy in comparison with those who only received TURBT, regardless of the other treatment modalities employed in the latter cases (chemotherapy, radiotherapy). Some studies tried to compare radical cystectomy with the chemo-radiotherapy treatment. The study of Haresh et al. concluded that the chemo-radiation yielded equivalent survival results with radical cystectomy. So, it is worth giving preference to chemo-radiation, because this strategy will lead to a better quality of life for the patient [**15**]. The same results were obtained by Kachnic et al., who obtained a 52% survival rate at 5 years by using a multimodality treatment, similar to that obtained with cystectomy in patients having similar age and disease stage [**16**]. What should be cleared out is that in those studies, the patients were treated with cystectomy followed by adjuvant chemotherapy or with neoadjuvant chemotherapy and concurrent chemo-radiotherapy. In our study, only 5% of the patients received concurrent chemo-radiotherapy with cisplatin alone, and only 9% neoadjuvant chemotherapy.

Increasing new data supported the role of neoadjuvant chemotherapy for T2 and T3 lesions [17-19]. Regimens used for our patients in neoadjuvant setting were MVAC and gemcitabine plus cisplatin, similar with those used in clinical trials [**20**]. In a recent meta-analysis of neoadjuvant trials, a statistically significant decrease in the death rate was seen, corresponding to an improvement in the overall survival (an estimated 9% at 5 years) [**21**], so it is expected that the percentage of patients receiving neoadjuvant chemotherapy would grow in the future. 

Of all the patients, 63.2% received chemotherapy as a single treatment or in combination with radiotherapy. In 20% of the cases, chemotherapy was administered in the adjuvant setting. The data regarding the adjuvant chemotherapy are conflicting. The results of currently available trials suggested that adjuvant chemotherapy could delay recurrences, which may justify the administration of chemotherapy in those at high risk of relapse. The most used regimens were MVAC and Gemcitabine plus Cisplatin [**22**,**23**,**24**,**25**]. 

Radiation alone is not considered a standard treatment for patients with an invasive bladder tumor. Because the initial complete response and long-term bladder preservation rates are higher with chemotherapy combined with radiotherapy, this is the preferred treatment. Because the results of radiotherapy alone were considered inferior to those of radical surgery, radiotherapy alone was only indicated for those who could not tolerate a cystectomy or chemotherapy because of medical comorbidities [**26**]. In our patients, the percent treated with radiotherapy alone was 36.8%. The majority of them were elderly patients (>70 years) who were unable to tolerate another treatment. 

In patients with a metastatic disease, the mainstay of treatment is chemotherapy. The standard chemotherapy for advanced bladder carcinoma has been MVAC for more than a decade. Based on several phase II and phase III trials, gemcitabine and cisplatin provided a similar survival advantage to MVAC with a better safety profile and tolerability [**25**,**27**]. In our study, the most used regimen was gemcitabine and cisplatin, due to a more favorable toxicity profile.

The limited survival and high relapse rate in urinary bladder cancer revealed the necessity of improving a surgical and oncologic approach. Benefits and risks of partial cystectomy as compared with total cystectomy should be clarified [**28**]. Also, a new regimen of chemotherapy should be tried for survival improving [**29**].

Our data regarding the outcome of patients with muscular invasive bladder carcinoma showed satisfactory results in terms of disease-free survival for stage II and III and progression-free survival for stage IV patients. The median survival in stage IV of the disease was not more than 14 month in any series [**27**]. The limitations of our study consisted in the lack of survival data (only recurrence or progression were documented in all patients) and the small follow-up interval. 

In conclusion, urinary bladder carcinoma is very aggressive and very often leads to death. The prognosis of these patients can be improved by an early diagnosis, radical surgery with curative intent (cystectomy), and adjuvant multimodal treatment (radiotherapy and chemotherapy). Neoadjuvant chemotherapy should play a more important role in the treatment of patients having bladder carcinoma with muscular invasion.
